# Parieto-occipital sulcus widening differentiates posterior cortical atrophy from typical Alzheimer disease

**DOI:** 10.1016/j.nicl.2020.102453

**Published:** 2020-09-28

**Authors:** Giorgio G. Fumagalli, Paola Basilico, Andrea Arighi, Matteo Mercurio, Marta Scarioni, Tiziana Carandini, Annalisa Colombi, Anna M. Pietroboni, Luca Sacchi, Giorgio Conte, Elisa Scola, Fabio Triulzi, Elio Scarpini, Daniela Galimberti

**Affiliations:** a Fondazione IRCCS Ca’ Granda Ospedale Maggiore Policlinico, via F. Sforza, 35, 20122 Milan, Italy; b Department of Neuroscience, Psychology, Drug Research and Child Health, University of Florence, 50121 Firenze, Italy; c Neurological Department, ASST Lecco, Lecco, Italy; d Department of Neurology, Amsterdam University Medical Centers, Location VUmc, Alzheimer Center, Amsterdam, the Netherlands; e Department of Pathophysiology and Transplantation, Dino Ferrari Center, University of Milan, Milan, Italy; f Department of Biomedical, Surgical and Dental Sciences, Dino Ferrari Center, CRC Molecular Basis of Neuro-Psycho-Geriatrics Diseases, University of Milan, Milan, Italy

**Keywords:** AD, Alzheimer disease, AT, Anterior temporal scale, AUC, Area under the ROC Curve, CON, Controls, CSF, Cerebrospinal Fluid, FDG, fluorodeoxyglucose, FEW, Family wise error, GM, grey matter, LBD, Lewy Body Dementia, MMSE, Mini Mental State Examination, MNI, Montreal Neurological Institute, MRI, magnetic resonance imaging, MTA, Medial temporal scale, OF, Orbito-Frontal scale, PA, Posterior scale, PCA, Posterior cortical atrophy, PCS, Posterior cingulate sulcus scale, PET, Positron emission tomography, POS, Parieto-occipital sulcus scale, PRE, Precuneus scale, VBM, Voxel Based Morphometry, VOSP, Visual object and space perception test, WM, white matter, Posterior Cortical Atrophy, Alzheimer Disease, Visual rating scale, Voxel based morphometry, Differential Diagnosis

## Abstract

**Objectives:**

Posterior Cortical Atrophy (PCA) is an atypical presentation of Alzheimer disease (AD) characterized by atrophy of posterior brain regions. This pattern of atrophy is usually evaluated with Koedam visual rating scale, a score developed to enable visual assessment of parietal atrophy on magnetic resonance imaging (MRI). However, Koedam scale is complex to assess and its utility in the differential diagnosis between PCA and typical AD has not been demonstrated yet. The aim of this study is therefore to spot a simple and reliable MRI element able to differentiate between PCA and typical AD using visual rating scales.

**Methods:**

15 patients who presented with progressive complex visual disorders and predominant occipitoparietal hypometabolism on PET-FDG were selected from our centre and compared with 30 typical AD patients and 15 healthy subjects. We used previously validated visual rating scales including Koedam scale, which we divided into three major components: posterior cingulate, precuneus and parieto-occipital. Subsequently we validated the results using the automated software Brainvisa Morphologist and Voxel Based Morphometry (VBM).

**Results:**

Patients with PCA, compared to typical AD, showed higher widening of the parieto-occipital sulcus, assessed both with visual rating scales and Brainvisa. In the corresponding areas, the VBM analysis showed an inverse correlation between the results obtained from the visual evaluation scales with the volume of the grey matter and a direct correlation between the same results with the cerebrospinal fluid volume.

**Conclusions:**

A visually based rating scale for parieto-occipital sulcus can distinguish Posterior Cortical Atrophy from typical Alzheimer disease.

## Highlights

• Parieto-occipital sulcus visual rating scale can distinguish PCA from typical AD.
• Visual rating scales have been validated using VBM and Brainvisa Morphologist.
• Visual rating score reflects sulcal widening rather than grey matter reduction.

## 1 Introduction

Posterior Cortical Atrophy (PCA) is a clinico-radiological syndrome mainly characterized by the loss of tissue in the posterior regions of the brain and the current diagnostic criteria consider the presence of MRI atrophy in these regions as a supportive feature (Crutch et al., 2017). The most frequent neuropathologic cause of PCA is Alzheimer disease (AD), but other, much rarer, alternative aetiologies have been identified such as Lewy body dementia (LBD), Corticobasal Degeneration and Creutzfeldt Jakob disease (Renner et al., 2004, Tang-Wai et al., 2003).

Visual rating scales have shown to be an effective and economic way to support the clinical diagnosis in differentiating between various form of dementia (Harper et al., 2015). In particular, the posterior scale developed by Koedam et al, validated by a pathologically confirmed cohort (Lehmann et al., 2012) and voxel-based morphometry (VBM) (Möller et al., 2014), have demonstrated to be able to differentiate between patient with typical AD and patients with subjective memory complains or other form of dementia (Koedam et al., 2011). Koedam’s scale takes in consideration 4 different areas (posterior cingulate sulcus, precuneus, parieto-occipital sulcus, and sulci in the parietal cortex), each of them needs to be evaluated on the three axes, as they can be differently atrophied. Therefore, the rater has to condense 4 different variables for each side (for a total of 8) in one single score. This approach shows some difficulties limiting its use: it is time consuming, it requires specific sequences and there are no clear indications regarding the slice selection except for the sagittal view. In the paper the authors state that “in cases of different scores on different orientation the highest score was considered” (Koedam et al., 2011), but it has subsequently been demonstrated that the medial region influences the scoring more than the other areas (Möller et al., 2014). Despite being designed to evaluate posterior atrophy in subjects with typical AD, there are no definitive studies on the use of the posterior scale in patients with PCA.

Brainvisa Morphologist is a recently developed software able to automatically reconstruct and identify cortical sulci, allowing to extract various parameters for each sulcus and particularly its width (Mangin et al., 2010). This automated approach has already been applied in typical AD patients, showing that fold opening of the parieto-occipital fissure and the intraparietal sulcus could be used as a biomarker, as they are able to differentiate between mild cognitive impairment, typical AD and controls (Hamelin et al., 2015, Plocharski and Østergaard, 2016, Reiner et al., 2012).

Previous studies used VBM to analyse pattern of grey matter (GM) in PCA showing predominant parieto-occipital atrophy (Agosta et al., 2018, Lehmann et al., 2011, Migliaccio et al., 2009). The main limitation of this tool is the poor applicability in the clinical practice since it performs a comparison between groups, it requires specific software and time-consuming analyses.

In this study, we simplified the Koedam posterior scale dividing it into three major parts, with the aim of identifying the best component able for the differentiation between Posterior Cortical Atrophy and typical AD. The utility of these new subscales in the differential diagnosis of PCA was thenvalidated through unbiased automatic methods (Brainvisa Morphologist and VBM).

## 2 Methods

### 2.1 Population

Forty-five patients (30 typical AD and 15 PCA) were recruited at the Neurodegenerative Diseases Unit of the Fondazione Ca’ Granda, IRCCS Ospedale Maggiore Policlinico from September 2014 to January 2018. Inclusion criteria for PCA were the current clinical criteria (insidious onset of visual and parietal functions with gradual progression with a least 3 symptoms of those explained in (Crutch et al., 2017)) and, in addition, the evidence of hypometabolism in occipito-parietal or occipito-temporal regions on Positron Emission Tomography with fluorodeoxyglucose (FDG-PET), in order to avoid redundancy in the use of MRI as an inclusion criteria. PCA patients were subsequently classified in PCA due to LBD (if they fulfilled at least two core criteria for LBD) (McKeith et al., 2017), PCA due to AD (not fulfilling two core criteria for LBD and with positive amyloid biomarkers) and PCA pure (those not fulfilling two core criteria for LBD and with negative amyloid biomarkers).

Typical AD patients were included when they met IGW-2 criteria (Dubois et al., 2014): progressive change in memory and evidence of amyloid deposition at Florbetapir-PET or levels of amyloid β in cerebrospinal fluid (CSF) below 660 pg/ml with a ratio total-tau/amyloid β above 0.34 (Irwin et al., 2012).

At last, 15 age and gender matched controls (CON) without cognitive deficits and with Mini mental state examination (MMSE) score equal or higher than twenty-seven were recruited for the study.

All the subjects underwent a general and neurological examination, detailed medical history and MMSE. Typical AD and PCA patients underwent a standardized neuropsychological battery that included: trail making test A, phonemic and semantic fluency, Raven coloured progressive matrices, digit span forward and backward, Corsi block tapping test, clock drawing test, Rey figure copy, denomination and logical memory. PCA patients also underwent visual object and shape perception test (VOSP) (Warrington and James, 1991). All patients underwent lumbar puncture or PET with Florbetapir. CSF Amyloid β 1–42, phospho-tau and total-tau were measured by using the commercially available sandwich enzyme-linked immunosorbent assay kits (Fujirebio, Ghent, Belgium). Florbetapir-PET data were first qualitatively analysed by a trained physiologist using a binary method of interpretation for relating “positive” or “negative” scans to neuropathologically defined categories of Amyloid β plaque density.

Exclusion criteria for this study included ophthalmologic disease, rapidly evolving dementia or substantial MRI T2 white matter hyperintensities in the occipito-parietal regions.

This study was approved by the Local Ethical Committee on human studies and written informed consent from all subjects was obtained prior to their enrolment.

### 2.2 MRI acquisition

The MRI was performed with a 3 Tesla scanner (Achieva, Philips Healthcare, Eindhoven, Netherlands) using a 32-channel phase-array head coil. Whole-brain tridimensional (3D) T1-weighted turbo field-echo sequence was acquired in the sagittal plane with the following parameters: repetition time = 10 ms, Echo time 7.9 ms, flip angle = 8°, field of view = 250 × 250 mm^2^, voxel size = 0.9 × 0.9 × 0.9 mm^3^. For clinical purpose the MR protocol also included 3D T2-weighted Fluid Attenuated Inversion Recovery (FLAIR) images, axial fast spin-echo T2-weighted images and axial diffusion-weighted.

### 2.3 MRI analysis

#### 2.3.1 Visual rating

**Fig. 1:**
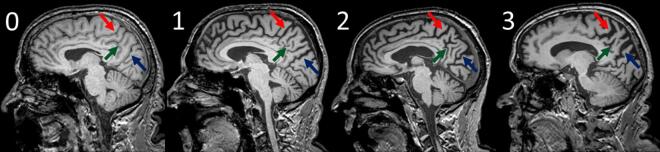
Reference images of Posterior atrophy: 0) closed sulci 1) mild widening 2) moderate widening 3) severe widening. Arrow indicate the sulci: red posterior cingulate sulcus (PCS), green precuneus (PRE), blue parieto-occipital sulcus (POS). (For interpretation of the references to color in this figure legend, the reader is referred to the web version of this article.)

A protocol of 4 different visual rating scales, as described in previously published papers (Fumagalli et al., 2018, Harper et al., 2016), was applied independently by two expert raters (GGF and PB, both clinical neurologists, after a training set of 15 scans), blinded for clinical and demographical information. In particular, the scales used were: Orbitofrontal (OF), Anterior Temporal (AT), Medial Temporal (MTA) and Koedam Posterior scale (PA). Briefly, the OF scale is rated in the coronal plane on the most anterior slice where the corpus callosum becomes visible with a four-part grading system: grade 0, representing no atrophy (no cerebrospinal fluid [CSF] visible within the sulcus); grade 1, mild widening of the sulcus (CSF just becomes visible); grade 2, moderate widening; and grade 3, severe widening (with the sulcus assuming a triangular shape). The AT scale looked at the aspects of temporal pole in coronal view, using a 5-point system: grade 0 representing normal appearances, grade 1 only slight prominence of anterior temporal sulci, grade 2 definite widening of the temporal sulci, grade 3 severe atrophy and ribbon-like nature of the gyri, and grade 4 a simple linear profile of the temporal pole. The MTA is a 5-point graded scale that looks at the medial temporal lobe in coronal view: grade 0 is normal; grade 1 a widened choroidal fissure; grade 2 an increased widening of the choroidal fissure, widening of temporal horn and opening of other sulci; grade 3 pronounced volume loss of the hippocampus; and grade 4 end-stage atrophy. PA scale is a 4-point scale evaluating posterior cortical atrophy using three views (coronal, axial and sagittal): grade 0 representing closed posterior cingulate and parieto-occipital sulci; grade 1 mild widening of the posterior cingulate and parieto-occipital sulci, with mild atrophy of the parietal lobes and precuneus; grade 2 substantial widening of the posterior cingulate and parietooccipital sulcus, with substantial atrophy of the parietal lobes and precuneus; and grade 3 end-stage atrophy with evident widening of both sulci and knife-blade atrophy of the parietal lobes and precuneus. Furthermore, the Posterior scale has been divided in three subscales evaluated in the sagittal view: the posterior cingulate sulcus (PCS), the precuneus (PRE) and the parieto-occipital sulcus (POS) (See Fig. 1). Right and left sides were assessed separately and for each subscale the raters were asked to give a score between 0, which represented a closed sulcus, to 3, which represented an evident widening of the sulcus. The scores obtained were then combined to obtain a mean score.

In order to increase rating consistency, reference images for each scale were provided (Fig. 1). Lastly, the raters re-rated a subset of 50 randomly chosen subjects to calculate intra-rater reliability. The software used to display images was MRIcron (Rorden et al., 2007); images has been rated in the native space, in keeping with standard clinical reads.

#### 2.3.2 Automated sulcal extraction

Brainvisa 4.5 Morphologist pipeline was used for the reconstruction of cortical sulci from T1 MRI images (http://brainvisa.info), as previously reported (Mangin et al., 2010). Concisely, MRI images were imported into the Brainvisa database, registered, bias corrected and lastly segmented into GM, white matter (WM), and CSF (Mangin et al., 2004). Then, brain sulci were automatically extracted and labelled (Rivière et al., 2002), calculating their widening: for both sides of each sulcus the fold widening (in millimetres) was automatically calculated as the mean distance between the two walls of the pial surface which defines the cortical sulci. Data have been visually checked at each step of the procedure.

We selected the sulci that corresponded to the visual rating scales used: olfactory sulcus for orbitofrontal scale (OF), temporal pole for anterior temporal scale (AT), posterior cingulate fissure for posterior cingulate sulcus (PCS), sub-parietal and internal parietal sulci for precuneus (PRE), parieto-occipital for parieto-occipital sulcus (POS) and a mean score of all the posterior scores for posterior scale (PA). Regarding medial temporal scale (MTA), where the score corresponds to the medial region of temporal lobe and not only to a single sulcus, we have chosen to analyse the most proximal sulcus extracted, in this case the collateral sulcus.

#### 2.3.3 Voxel based morphometry

In order to explore the relationship between each rating scale and the volume of GM and CSF, VBM analysis was performed using Statistical Parametric Mapping 12 (http://www.fil.ion.ucl.ac.uk/spm). T1-weighted images were normalized and segmented into GM, WM and CSF probability maps using standard procedures and the fast-diffeomorphic image registration (DARTEL) algorithm (Ashburner, 2007). GM and CSF segments were affine-transformed into the Montreal Neurological Institute (MNI) space, then, before the analysis, modulated and smoothed using a Gaussian kernel with 6-mm full-width half-maximum (FWHM). In order to identify potential outliers, final smoothed-modulated-warped GM images were checked for sample homogeneity using CAT12 toolbox.

The GM and CSF tissue maps were fitted to a multiple regression model with the aim of identifying correlations with the visual rating scales (OF, AT, MTA, PA, PCS, PRE, POS). Age, gender and total intracranial volume were entered as covariates. Group comparison was made on a voxel-level using two-sample t-tests. To highlight only areas that could have clinical utility the significance threshold was set at 0.05 corrected for multiple comparison (family-wise error) when comparing groups of patients with controls and at 0.001 cluster level corrected when comparing PCA with typical AD.

### 2.4 Statistical analysis

The program used for the statistical analysis was SPSS version 22. Group differences has been tested using *t* test for age, education, MMSE and neuropsychological tests, chi squared for gender and Mann-Whitney *U* test for visual rating and Brainvisa scores as they failed Shapiro-Wilk test for normal distribution. Area under the Receiver operating characteristic curve (AUC) was calculated for each significant comparison. For intra and inter-rater agreement weighted Kappa has been calculated. The correlations were analysed with Spearmann rank correlation.

## 3 Results

### 3.1 Demographic

All three groups were comparable in terms of age, education and gender distribution (See Table 1). The PCA and typical AD groups were also comparable in terms of MMSE (18.27 ± 4.67 and 19.21 ± 5.43 respectively). Regarding neuropsychological testing AD patients scored lower in logical memory test while PCA patients were lower in Corsi block tapping test and Rey figure copy (see Table 1).

**Table 1:** Demographic data and mean scores of neuropsychological testing.

	Subjects			Statistical significance		
	Typical AD	PCA	CON	tAD-PCA	PCA-CON	tAD-CON
Number	30	15	15			
Age	68.93 (7.56)	68.76 (7.37)	69.51 (6.52)	n.s.	n.s.	n.s.
Gender	22F 8M	8F 7M	7F 8M	n.s.	n.s.	n.s.
Education	8.72 (3.57)	9.27 (3.83)	7.78 (3.27)	n.s.	n.s.	n.s.
MMSE	19.55 (5.3)	18.27 (4.67)	29.25 (0.96)	n.s.	<0.01	<0.01
Raven coloured progressive matrices	30.57 (14.21)	25.50 (11.98)		n.s.		
Trail Making Test A	116.47 (89.42)	211.67 (88.08)		n.s.		
Phonemic fluency	18.79 (9.45)	17.91 (8.71)		n.s.		
Semantic fluency	19.92 (11.68)	19.36 (9.34)		n.s.		
Digit span Forward	4.50 (1.18)	4.07 (0.62)		n.s.		
Digit span Backward	2.88 (0.60)	2.55 (0.69)		n.s.		
Corsi block tapping test	3.43 (0.73)	2.50 (1.35)		<0,01		
Clock drawing test	3.59 (3.07)	2.38 (2.33)		n.s.		
Logical memory	0.72 (1.82)	3.44 (2.86)		<0,01		
Rey figure copy	18.68 (11.54)	1.83 (3.73)		<0,01		
Denomination	38.50 (22.20)	36.64 (17.24)		n.s.		

Results presented are mean scores (Standard Deviation) for Subjects. Statistical significance is chi-squared for gender and *t*-test for age, education and neuropsychological tests. Abbreviations: tAD typical Alzheimer patients PCA Posterior cortical atrophy patients CON healthy control subjects; MMSE mini mental state examination, n.s. non-significant difference.

According to current criteria (Crutch et al., 2017), among PCA patients, 10 subjects had a diagnosis of PCA due to AD, 3 subjects PCA plus Lewy Body Dementia and 2 subjects pure PCA (see Table 2).

**Table 2:** Characteristics of PCA patients.

	PCA subject number	PCA 1	PCA 2	PCA 3	PCA 4	PCA 5	PCA 6	PCA 7	PCA 8	PCA 9	PCA 10	PCA 11	PCA 12	PCA 13	PCA 14	PCA 15
	Diagnosis	PCA-AD	PCA-AD	PCA-AD	PCA-AD	PCA-AD	PCA-AD	PCA-AD	PCA-AD	PCA-AD	PCA-AD	PCA pure	PCA pure	PCA-LBD	PCA-LBD	PCA-LBD

CSF	Amyloid β	307	601	375	410	397	353	566	644	307	540	738	718		1231	552
	Total Tau	337	793	491	194	219	392	705	393	546	1324	342	939		258	207
	Phospho Tau	54	48	48	31	46	51	84	62	88	133	64	115		53	43
	Total Tau/Amyloid β ratio	1,10	1,32	1,31	0,47	0,55	1,11	1,25	0,61	1,78	2,45	0,46	1,31		0,21	0,38

Amyloid PET	Florbetapir PET				POS				POS	POS				NEG	NEG	

Lewy body dementia core clinical criteria	Fluctuations														x	x
	Visual hallucinations						x					x		x	x	
	Parkinsonism													x		x
	REM sleep disorder		x													

PCA clinical criteria	Space perception deficit					x			x	x		x	x		x	x
	Simultanagnosia	x		x			x		x	x		x	x			x
	Object perception deficit	x	x	x		x	x	x	x				x	x	x	
	Constructional dyspraxia		x	x	x			x	x		x		x	x	x	x
	Environmental agnosia						x		x	x	x		x		x	
	Oculomotor apraxia				x	x	x						x			
	Dressing apraxia		x				x	x								
	Optic ataxia				x	x	x					x	x			
	Alexia				x				x	x						x
	Left/right disorientation										x					
	Acalculia					x			x		x	x	x	x		
	Limb apraxia		x									x		x		
	Prosopagnosia	x					x						x	x		
	Agraphia				x			x	x	x	x		x	x		x
	Homonymous visual field defect	x			x	x							x	x		x
	Finger agnosia				x		x			x	x		x			

Visual Object and Space Perception	Shape detection screening test	pass	fail	pass	pass	fail	pass	pass	fail	pass	pass	pass	pass	fail	pass	pass
	Incomplete letters	19		10*	11*		0*	0*		1*	2*	3*	2*		14*	5*
	Silhouettes	23		6*	4*		6*	3*		7*	7*	9*	4*		11*	8*
	Object decision	19		6*	12*		15	8*		8*	3*	8*	3*		19	18
	Progressive silhouettes	10*		1*	1*		4*	0*		11*	0*	2*	4*		12*	6*
	Dot counting	10		9	0*		6*	2*		9	3*	10	4*		8*	3*
	Position Discrimination	9*		14*	10*		14*	7*		13*	2*	13*	8*		18*	7*
	Number location	3*		10	1*		4*	3*		0*	2*	9	4*		8	3*
	Cube analysis	5*		3*	0*		0*	0*		1*	3*	5*	3*		7	4*

Biological, clinical and cognitive characteristics of PCA patients.

Inclusion criteria for PCA were at least 3 symptoms of visual and parietal functions. Subjects were subsequently classified in PCA due to LBD if they fulfilled at least two core criteria for LBD (McKeith et al., 2017), PCA due to AD if not fulfilled at least two core criteria for LBD and with positive amyloid biomarkers and PCA pure if did not fulfilled at least two core criteria for LBD and with negative amyloid biomarkers. x represents when a symptom is present.

Four patients failed at the shape detection screening test of Visual object and space perception while eleven completed all the tests. Data are raw scores with * representing a pathological result.

Abbreviations: POS positive; NEG negative.

In our sample 27 out of 30 patients with typical AD underwent lumbar puncture while three subjects had only an Amyloid PET positivity. All the subjects with CSF value available had Amyloid β levels below 660 pg/ml and all of them had a ratio T-Tau/Amyloid β above 0.34. Among them 5 with Amyloid β > 600 pg/ml and <660 pg/ml also had an Amyloid PET positivity.

### 3.2 Visual rating scales

#### 3.2.1 Inter-intra rater

All the scales have demonstrated good inter-rater reliability with weighted Kappa score higher than 0.62, and the MTA scale performed best overall (Table 3). Considering the intra-rater scores, rater 1 weighted Kappa were greater than 0.79 for all the scales, and rater 2 had weighted Kappa scores greater than 0.77.

**Table 3:** Inter- and Intra-rater agreement scores and correlation between visual rating and Brainvisa Morphologist.

	Inter-rater	Intra-rater		Correlation
Visual rating scale		Rater 1	Rater 2	Visual-Brainvisa
OF Orbitofrontal	0.75	0.89	0.80	0.37
AT Anterior temporal	0.64	0.79	0.77	0.60
MTA Medial temporal	0.85	0.92	0.84	0.40
PA Posterior atrophy	0.69	0.92	0.84	0.81
PCS Posterior cingulate	0.74	0.86	0.83	0.73
PRE Precuneus	0.62	0.84	0.80	0.70
POS Parieto-occipital	0.68	0.88	0.82	0.77

Inter- and intra-rater agreement scores are the weighted Kappa, correlation is the correspondent Spearmann rank correlation coefficient. Abbreviations: OF Orbitofrontal rating scale (olfactory sulcus), AT Anterior temporal rating scale (temporal pole), MTA Medial temporal atrophy rating scale (collateral sulcus), PA Posterior atrophy rating scale (posterior cingulate, sub parietal and internal parietal and parieto-occipital sulci), PCS Posterior cingulate atrophy rating scale (posterior cingulate sulcus), PRE precuneus atrophy rating scale (sub parietal and internal parietal sulci), POS parieto-occipital rating scale (parieto-occipital sulcus).

#### 3.2.2 Visual rating scales, comparison between groups

Results are summarized in Table 4. Typical AD patients scored significantly higher than CON in all visual rating scales except for POS, obtaining the highest AUC in the AT and MTA scales. PCA patients had higher scores in all the scales except for OF scale, obtaining the highest AUC in the MTA scale. The comparison between PCA and typical AD patients demonstrated that only the POS scale was significantly higher in the PCA subjects with an AUC of 0.74.

**Table 4:** Mean scores of sulcal widening with visual rating scales and with Brainvisa morphologist.

Scale	Method	Subjects			Statistical significance		
		tAD	PCA	CON	tAD-PCA	PCA-CON	tAD-CON
OF	Visual rating	0.94 (0.76)	0.80 (0.62)	0.38 (0.48)	n.s.	n.s.	<0.01 (0.73)
	*Brainvisa*	*2.17 (0.76)*	*1.91 (0.38)*	*1.59 (0.33)*	*n.s.*	*<0.01 (0.76)*	*<0.01 (0.77)*

AT	Visual rating	1.23 (0.70)	1.03 (0.59)	0.38 (0.31)	n.s.	<0.01 (0.82)	<0.01 (0.86)
	*Brainvisa*	*2.64 (0.77)*	*2.43 (0.88)*	*1.65 (0.60)*	*n.s.*	*<0.01 (0.76)*	*<0.01 (0.85)*

MTA	Visual rating	1.55 (0.91)	1.48 (0.88)	0.43 (0.38)	n.s.	<0.01 (0.85)	<0.01 (0.86)
	*Brainvisa*	*1.46 (0.70)*	*2.05 (0.86)*	*0.71 (0.29)*	*<0.05 (0.70)*	*<0.01 (0.96)*	*<0.01 (0.86)*

PA	Visual rating	1.38 (0.77)	1.80 (0.88)	0.75 (0.70)	n.s.	<0.01 (0.83)	<0.01 (0.73)
	*Brainvisa*	*2.08 (0.83)*	*2.29 (0.67)*	*1.39 (0.44)*	*n.s.*	*<0.01 (0.87)*	*<0.01 (0.82)*

PCS	Visual rating	1.58 (0.76)	1.72 (0.93)	0.92 (0.72)	n.s.	<0.01 (0.76)	<0.01 (0.73)
	*Brainvisa*	*2.58 (0.83)*	*2.37 (0.87)*	*1.80 (0.64)*	*n.s.*	*n.s.*	*<0.01 (0.79)*

PRE	Visual rating	1.28 (0.72)	1.58 (0.89)	0.73 (0.60)	n.s.	<0.01 (0.79)	<0.05 (0.71)
	*Brainvisa*	*1.93 (0.63)*	*2.14 (0.70)*	*1.20 (0.46)*	*n.s.*	*<0.01 (0.86)*	*<0.01 (0.82)*

POS	Visual rating	1.09 (0.71)	1.73 (0.89)	0.38 (0.75)	<0.01 (0.74)	<0.01 (0.80)	n.s.
	*Brainvisa*	*1.89 (0.58)*	*2.49 (0.79)*	*1.36 (0.44)*	*<0.01 (0.73)*	*<0.01 (0.88)*	*<0.01 (0.76)*

Results presented are mean scores (Standard Deviation) for Subjects and Mann-Whitney U (Area under the Curve) for Statistical significance. Abbreviations: tAD typical Alzheimer patients, PCA Posterior cortical atrophy patients, CON healthy control subjects; OF Orbitofrontal rating scale (olfactory sulcus), AT Anterior temporal rating scale (temporal pole), MTA Medial temporal atrophy rating scale (collateral sulcus), PA Posterior atrophy rating scale (posterior cingulate, sub parietal and internal parietal and parieto-occipital sulci), PCS Posterior cingulate atrophy rating scale (posterior cingulate sulcus), PRE precuneus atrophy rating scale (sub parietal and internal parietal sulci), POS parieto-occipital rating scale (parieto-occipital sulcus).

### 3.3 Brainvisa, comparison between groups

Typical AD patients had significantly wider sulci than CON in all sulci analysed with the highest AUC for the collateral sulcus (Table 4). PCA patients had significantly wider sulci than CON except for the posterior cingulate sulcus, showing the highest AUC in the collateral sulcus. Compared to typical AD patients, PCA patients had wider parieto-occipital (AUC 0.73) and collateral sulci (AUC 0.70).

### 3.4 Voxel based morphometry, comparison between groups

#### 3.4.1 PCA-CON

**Fig. 2:**
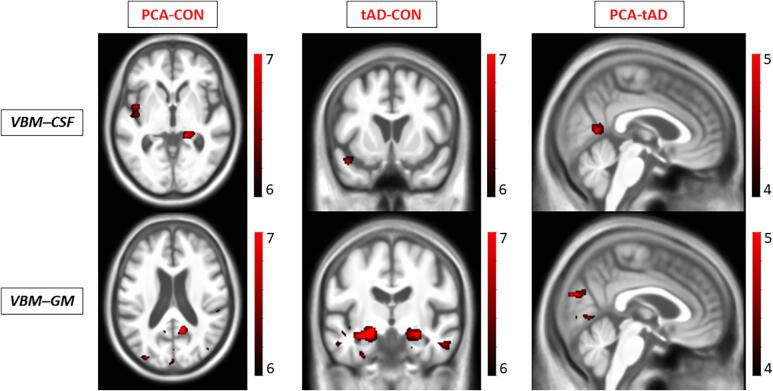
Voxel based morphometry comparison between groups. Top row VBM study on increase of CSF, bottom row VBM study on GM atrophy. There was no evidence of greater GM loss or CSF increases in typical AD relative to PCA or controls relative to either patient group. Results are overlaid on a study-specific template in MNI (Montreal Neurological Institute) space. Images are shown with the left hemisphere on the left side of the figure and displayed at p < 0.05 family wise error (FWE) corrected for multiple comparisons for the contrast PCA-CON and AD-CON while cluster level corrected at p < 0.01 false discovery rate (FDR) for the contrast PCA-AD. Color bar represent the Z score. Abbreviations: tAD typical Alzheimer patients; PCA Posterior cortical atrophy patients; CON healthy control subjects.

The PCA group showed GM loss at the level of right precuneus, posterior cingulate bilaterally and right fusiform area (Fig. 2). The most significant loss involved the superior parietal area (Brodmann area 7). The PCA group also showed two regions of increased CSF volume: one near the right ventricle and one near the left insula. No regions showed GM loss or CSF increase in the comparison between control subjects and PCA subjects (supplementary table).

#### 3.4.2 Typical AD-CON

Compared to controls, the typical AD group presented a pattern of GM loss involving different areas: hippocampus bilaterally, right fusiform, posterior cingulate bilaterally, left inferior temporal and right middle temporal and left orbitofrontal. Typical AD group showed an increase of CSF volume in a region near the left temporal pole when compared to the control group. No regions showed greater GM loss or CSF increase in the control group than the typical AD group.

#### 3.4.3 PCA-typical AD

PCA patients compared to typical AD patients presented significantly greater atrophy in the right visual associative area (Brodmann areas 18–19) and right fusiform area (Brodmann area 37) (Cluster level corrected p < 0.001). The PCA group also showed an increased CSF volume in visual associative regions bilaterally (Brodmann area 18) and in right posterior cingulate (Brodmann area 23) (Cluster level corrected p < 0.001). No region showed greater GM loss or CSF increase in the typical AD group compared to PCA.

### 3.5 Correlations

#### 3.5.1 Correlation visual rating scales-Brainvisa

The correlation between the scores obtained from the visual rating and the correspondent sulcal widening observed with the automated tool was significant for all the sulci analysed with the highest correlation for PA scale (Table 3).

#### 3.5.2 Correlation visual rating scales-VBM

**Fig. 3:**
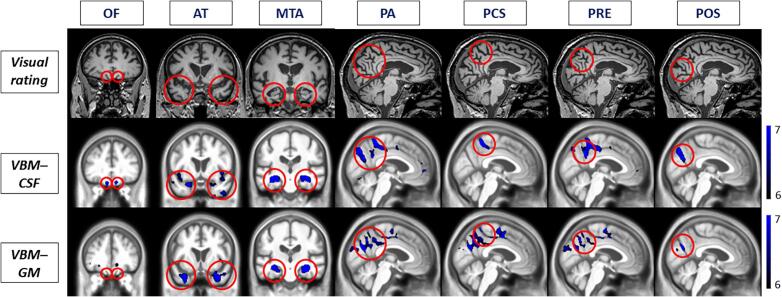
Visual rating scales and Voxel based Morphometry correlation analysis. In the first row the images represent an example subject while the second and third row represent a study specific mean template in MNI (Montreal Neurological Institute) space made with all the subjects. Red circles surround the area evaluated by the individual visual rating scales. Blue areas represent the Z score of the correlation between the score of the corresponding visual rating with CSF (second row, direct correlation) and GM volume (third row, inverse correlation) using SPM. Images are shown with the left hemisphere on the left side of the figure and displayed at p < 0.05 family wise error (FWE) corrected for multiple comparisons. Color bar represent the Z score. Abbreviations: OF Orbitofrontal rating scale (olfactory sulcus), AT Anterior temporal rating scale (temporal pole), MTA Medial temporal atrophy rating scale (collateral sulcus), PA Posterior atrophy rating scale (posterior cingulate, sub parietal and internal parietal and parieto-occipital sulci), PCS Posterior cingulate atrophy rating scale (posterior cingulate sulcus), PRE precuneus atrophy rating scale (sub parietal and internal parietal sulci), POS parieto-occipital rating scale (parieto-occipital sulcus). (For interpretation of the references to color in this figure legend, the reader is referred to the web version of this article.)

The voxel-based morphometric analysis revealed an inverse correlation of the scores from each visual rating scale score, identifying an area of GM atrophy and a direct CSF volume increase in the same expected region (Fig. 3).

No direct correlation for GM or inverse correlation for CSF was found.

#### 3.5.3 Correlation visual rating scales-neuropsychological tests

By calculating the correlation of neuropsychological scores of tAD and PCA patients with visual rating scales we found that digit span forward was inversely correlated with PAM (Spearman rho = −0.33), Corsi block tapping and denomination test were inversely correlated with OF (r = −0.35 and r = −0.38, respectively), whereas trail making test A was directly correlated with MTA (r = 0.42).

In PCA patients, VOSP number location was inversely correlated with PA (r = −0.81), PCS (r = −0.70), PRE (r = −0.85) and POS (r = −0.67) while VOSP position discrimination was inversely correlated with PCS (r = −0.67).

#### 3.5.4 Correlation Brainvisa-neuropsychological tests

Between Brainvisa and neuropsychological scores we found significant inverse correlations between OF and MMSE (r = −0.33), AT and digit span backwards (r = −0.47), MTA and MMSE (r = −0.37), Raven matrices (r = −0.36), digit span backwards (r = −0.69), clock drawing test (r = −0.34), Rey figure copy (r = −0.53) and direct correlation between MTA and logical memory (r = 0.4). PAM was inversely correlated with digit span forward (r = −0.31), digit span backwards (r = −0.52) and Rey figure copy (r = −0.37). PRE was inversely correlated with digit span backwards (r = −0.56) while POS with digit span forward (r = −0.33) and Rey figure copy (r = −0.38).

In PCA patients VOSP object decision was inversely correlated with AT (r = 0.76), VOSP number locations with PA (r = −0.76), PRE (r = −0.75) and POS (r = −0.72), VOSP cube analysis with PRE (r = −0.61) while VOSP progressive silhouettes with PCS (r = −0.65).

## 4 Discussion

In the present study we showed the presence of regional differences of cortical morphology between PCA, typical AD and CON.

Within the VBM differences in the grey matter between PCA and typical AD, the main one was the visual associative cortex, more pronounced on the right. These results are consistent with previous data (Agosta et al., 2018, Lehmann et al., 2012, Migliaccio et al., 2009, Ossenkoppele et al., 2015, Whitwell et al., 2007). We demonstrated that it is possible to spot those differences also with visual rating scales, with CSF VBM and with Brainvisa.

In a recent study that compared PCA due to AD from PCA due to other diseases it has been demonstrated that the first group had specific grey matter atrophy in the right dorsolateral prefrontal and medial temporal regions (Montembeault et al., 2018). Due to insufficient statistical power, we were not able to compare these two groups in our sample. To the best of our knowledge only one study has demonstrated higher scores for PCA in PA visual rating scale compared to typical AD, although this study was done with a small number of patients (Wang et al., 2015).

The PA scale proved to be highly correlated with the results obtained with Brainvisa, also demonstrating a high level of intra and inter rater agreement. Moreover, the PA scale was useful in the comparison between healthy subjects and patients of both conditions, but it did not differentiate between PCA and typical AD.

In their study Moeller et al compared groups of patients that had the same PA score (0 vs 1, 1 vs 2/3) demonstrating that different scores corresponded to different degrees of atrophy in VBM (Möller et al., 2014). Instead of comparing groups, we preferred to run a correlation analysis to obtain a more tailored correspondence with the area observed (Fig. 3), as already been shown in previous studies(Fumagalli et al., 2018, Harper et al., 2016).

In our opinion the visual rating score reflects more the widening of a sulcus (and consequently an increase in CSF) rather than a reduction of grey matter. The increase in CSF within a sulcus may be due to atrophy and morphological alterations not only of the grey matter but also of the white matter. The CSF increase may be an indirect way to calculate sulcal widening, that could be more accurate than GM atrophy.

We tested the correlation of visual rating scales with GM and with CSF separately, showing that the significant area reflected the observed area better for CSF than GM in all the scales except for the AT (Fig. 3).

All the three subscales of posterior atrophy (PCS, PRE, POS) had high level of intra-rater agreement and good level of inter rater. Moreover, all of them showed a very good correlation with the results obtained with Brainvisa and VBM probably because the sulci are evaluated on its entire length, and not only on part of it. With the limit of having tested only symptomatic patients, it is interesting to note that only digit span forward and VOSP number location correlated with posterior atrophy both with visual rating and Brainvisa. These results need further replications in larger cohorts that include also control subjects.

The POS scale proved to be useful in the differentiation between PCA and AD. The extent of the enlargement of the parieto-occipital sulcus calculated with Brainvisa for typical AD and CON is in line with a previous published study (Reiner et al., 2012), with PCA scoring higher in our analysis. The VBM analysis, especially the CSF one, confirmed that the widening of the parieto-occipital sulcus is a distinctive feature of PCA. Even though this has been demonstrated at a group level, further studies with the population stratified for severity are needed to identify early signs of the disease. Occipital and parietal lobes proved to be affected early in the disease in a recent work on longitudinal observation of PCA (Firth et al., 2019).

It is important to note that while the scores of PCS scale were higher in PCA than typical AD, the widening calculated with Brainvisa was higher in typical AD compared to PCA although not significant. This result can partially be explained by the different area analysed by the two methods: the visual rating assesses only the portion of the sulcus dividing the parietal from the frontal lobe, while Brainvisa evaluates that portion plus half of the cingulate sulcus.

Using single scales for the posterior sulci rather than Koedam scale reduces the time of assessment since they are evaluated only in sagittal view (therefore not requiring a 3D scan or an axial FLAIR as used in the original paper) and with precise anatomical landmarks it is easier to find the correct slice. The rater also does not need to decide how to weight the atrophy in each area for the final score.

As expected, the scores of OF scale were higher in typical AD compared to controls, but differences between typical AD and PCA did not reach the significance threshold. This scale showed lower correlation with Brainvisa scores than other scales, partly because the sample of typical AD and PCA patients had less frontal atrophy compared to other diseases (i.e. Frontotemporal dementia) and because of the longer extension of the olfactory sulcus compared to the small area that is tested with the visual scale.

The visual rating scale of medial temporal atrophy, as described in the original formulation, is a score representing the width of choroid fissure and temporal horn and the height of the hippocampal formation (Scheltens et al., 1992). Unfortunately these structures are not directly studied with the Brainvisa method. Considering this, we decided to use the collateral sulcus because of its proximity with the medial temporal area, but we didn’t find a high correlation with the visual rating scores. Previous studies reported the same problem, also due to the proximity of air-bone-brain interface and the lower contrast between grey and white matter (Reiner et al., 2012, Zhan et al., 2009). However, the widening of the collateral sulcus calculated with Brainvisa proved to be larger in PCA than typical AD. Since this sulcus extends into occipital lobe it’s widening might reflect atrophy in this area and therefore should be studied further.

The strengths of this study are its naturalistic approach, with images evaluated in native space, and the validation with two different automated methods. Visual rating scale are designed to be applied in the clinical context and have proved to be useful beyond being an economic, quick and easy to learn method.

The main limitation of the study is the small sample size, therefore it should be considered as preliminary and need replication. However, the subjects were well characterized even if not at a neuropathological level. For the clinical use, the method should be further tested in a single subject setting after the group comparison.

In conclusion widening of Parieto-occipital sulci by using visual rating scales can differentiate between Posterior cortical atrophy and typical AD. Visual rating scales have been validated with automated sulcal analysis and with VBM.

## CRediT authorship contribution statement

**Giorgio G. Fumagalli:** Conceptualization, Data curation, Formal analysis, Investigation, Methodology, Writing - original draft. **Paola Basilico:** Formal analysis, Investigation. **Andrea Arighi:** Data curation, Formal analysis. **Matteo Mercurio:** Data curation. **Marta Scarioni:** Data curation. **Tiziana Carandini:** Data curation. **Annalisa Colombi:** Data curation. **Anna M. Pietroboni:** Data curation. **Luca Sacchi:** Data curation. **Giorgio Conte:** Formal analysis. **Elisa Scola:** Formal analysis. **Fabio Triulzi:** Writing - review & editing. **Elio Scarpini:** Writing - review & editing. **Daniela Galimberti:** Writing - review & editing.

## Declaration of Competing Interest

The authors declare that they have no known competing financial interests or personal relationships that could have appeared to influence the work reported in this paper.
