# Supplementary material for: Parieto-occipital sulcus widening differentiates posterior cortical atrophy from typical Alzheimer disease
Source: Neuroimage Clin. 2020 Sep 28;28:102453. doi: 10.1016/j.nicl.2020.102453 (PMC7559336; doi:10.1016/j.nicl.2020.102453)
Supplement: Supplementary data 1 [file mmc1.docx]

| Location of the voxel showing significant GM loss on VBM in each of the group comparisons | | | | | | | | | |
| --- | --- | --- | --- | --- | --- | --- | --- | --- | --- |
| Comparison | Region (Brodmann area) | Side | X | Y | Z | Z score | Cluster size | P value | Correction |
| PCA vs tAD | Visual associative cortex (18) | R | 8 | -82 | 25 | 4.67 | 1634 | <0.001 | Cluster level |
|  | Fusiform gyrus (37) | R | 26 | -66 | -11 | 4.51 | 807 | 0.001 | Cluster level |
|  | Visual associative cortex (19) | R | 50 | -81 | 7 | 4.11 | 724 | 0.002 | Cluster level |
| tAD vs CON | Hippocampus (54) | L | -22 | -13 | 17 | 6.7 | 3490 | <0.001 | FWE |
|  | Fusiform gyrus (37) | R | 59 | -55 | 4 | 6.43 | 692 | <0.001 | FWE |
|  | Posterior cingulate cortex (23) | R | 14 | -54 | 19 | 6.41 | 983 | <0.001 | FWE |
|  | Inferior temporal gyrus (20) | L | -54 | -37 | -21 | 6.21 | 814 | <0.001 | FWE |
|  | Hippocampus (54) | R | 27 | -10 | -17 | 6.21 | 1807 | <0.001 | FWE |
|  | Orbitofrontal cortex(11) | L | -16 | 18 | -15 | 6.16 | 282 | <0.001 | FWE |
|  | Orbitofrontal cortex (11) | L | -21 | 53 | -12 | 5.98 | 290 | <0.001 | FWE |
|  | Middle temporal gyrus (21) | R | 57 | -36 | 3 | 5.93 | 482 | <0.001 | FWE |
|  | Middle temporal gyrus (21) | R | 56 | -10 | -27 | 5.83 | 265 | <0.001 | FWE |
|  | Posterior cingulate cortex (23) | L | -9 | -49 | 16 | 5.74 | 419 | <0.001 | FWE |
|  | Fusiform gyrus (37) | R | 42 | -37 | -20 | 5.58 | 96 | <0.001 | FWE |
| PCA vs CON | Precuneus (7) | R | 11 | -70 | 34 | 6.12 | 142 | <0.001 | FWE |
|  | Posterior cingulate cortex (23) | R | 12 | -55 | 18 | 6.1 | 111 | <0.001 | FWE |
|  | Posterior cingulate cortex (31) | L | -65 | -31 | -14 | 5.89 | 83 | <0.001 | FWE |
|  | Fusiform gyrus (37) | R | 44 | -39 | -20 | 5.65 | 87 | <0.001 | FWE |
| Location of the voxel showing the significant CSF increase on VBM in each of the group comparisons | | | | | | | | | |
| Comparison | Region (Brodmann area) | Side | X | Y | Z | Z score | Cluster size | P value | Correction |
| PCA vs tAD | Visual associative (18) | R | 3 | -60 | 9 | 4.37 | 1023 | <0.001 | Cluster level |
|  | Visual associative (18) | L | -26 | -100 | 6 | 4.54 | 619 | 0.001 | Cluster level |
|  | Posterior cingulate (23) | R | 24 | -57 | 6 | 3.57 | 540 | 0.002 | Cluster level |
| tAD vs CON | Temporal pole (38) | L | -45 | 12 | -23 | 5.37 | 39 | 0.002 | FWE |
| PCA vs CON | Outside BA Right ventricle | R | 17 | -34 | 0 | 5.53 | 182 | <0.001 | FWE |
|  | Insula (13) | L | -43 | 0 | 0 | 5.53 | 52 | <0.001 | FWE |

*Supplementary Table*  **Location of significant voxels on the VBM group comparison of GM and CSF**.

Coordinates (x, y, z) are in Montreal Neurological Institute space. Abbreviations: L=left; R=right, tAD typical Alzheimer patients PCA Posterior cortical atrophy patients CON healthy control subjects.
